# Differential Secretion of Angiopoietic Factors and Expression of MicroRNA in Umbilical Cord Blood from Healthy Appropriate-For-Gestational-Age Preterm and Term Newborns—*in Search of Biomarkers of Angiogenesis-Related Processes in Preterm Birth*

**DOI:** 10.3390/ijms21041305

**Published:** 2020-02-14

**Authors:** Dorota Gródecka-Szwajkiewicz, Zofia Ulańczyk, Edyta Zagrodnik, Karolina Łuczkowska, Dorota Rogińska, Miłosz P. Kawa, Iwona Stecewicz, Krzysztof Safranow, Bogusław Machaliński

**Affiliations:** 1 Department of General Pathology, Pomeranian Medical University, 70-111 Szczecin, Polandkawamilosz@gmail.com (M.P.K.);; 2 Department of Biochemistry and Medical Chemistry, Pomeranian Medical University, 70-111 Szczecin, Poland

**Keywords:** appropriate-for-gestational-age preterm newborns, angiopoietic factors, umbilical cord blood, peripheral blood, microRNA, angiogenesis, preterm gestation, prematurity, pro-angiogenic factors, angiostatic factors

## Abstract

**Objectives:** Premature birth, defined as less than 37 weeks gestation, affects approximately 12% of all live births around the world. Advances in neonatal care have resulted in the increased survival of infants born prematurely. Although prematurity is a known risk factor for different cardiovascular diseases, little is known about the pathophysiology of vasculature during premature gestation and angiopoietic factors network during premature birth. Aims: The objective of this study was to determine whether the profile of several pro-angiogenic and anti-angiogenic factors in umbilical cord blood (UCB) is different in healthy appropriate-for-gestational-age preterm newborns and normal term babies. The second aim of this study was to investigate the microRNA (miRNAs) expression profile in UCB from preterm labor and to detect miRNAs potentially taking part in control of angogenesis-related processes (Angio-MiRs). **Methods:** Using an immunobead Luminex assay, we simultaneously measured the concentration of Angiogenin, Angiopoietin-1, FGF-acidic, FGF-basic, PDGF-aa, PlGF, VEGF, VEGF-D, Endostatin, Thrombospondin-2, NGF, BDNF, GDNF, and NT-4 in UCB samples collected from the preterm (n = 27) and term (n = 52) delivery. In addition, the global microRNA expression in peripheral blood mononuclear cells (PBMCs) circulating in such UCB samples was examined in this study using microarray MiRNA technique. **Results:** The concentrations of five from eight measured pro-angiogenic factors (VEGF, Angiopoietin-1, PDGF-AA, FGF-a, and FGF-b) were significantly lower in UCB from preterm newborns. On the contrary, two angiostatic factors (Endostatin and Thrombospondin-2) were significantly up-regulated in preterm UCB. Among analyzed neurotrophins in preterm newborns, the elevated UCB concentration was found only in the case of GDNF, whereas BDNF was significantly reduced. Moreover, two angiopoietic factors, VEGF-D and PlGF, and two neurotrophins, NT4 and NGF, did not differ in concentration in preterm and term babies. We also discovered that among the significantly down-regulated miRNAs, there were several classical Angio-MiRs (inter alia MiR-125, MiR-126, MiR-145, MiR-150, or MiR155), which are involved in angiogenesis regulation in newborn after preterm delivery. **Conclusions:** This is the first report of simultaneous measurements of several angiopoietic factors in UCB collected from infants during preterm and term labor. Here, we observed that several pro-angiogenic factors were at lower concentration in UCB collected from preterm newborns than term babies. In contrast, the two measured angiostatic factors, Endostatin and Thrombospondin-2, were significantly higher in UCB from preterm babies. This can suggest that distinct pathophysiological contributions from differentially expressed various angiopoietic factors may determine the clinical outcomes after preterm birth. Especially, our angiogenesis-related molecules analysis indicates that preterm birth of healthy, appropriate-for-gestational-age newborns is an “anti-angiogenic state” that may provide an increased risk for improper development and function of cardiovascular system in the adulthood. This work also contributes to a better understanding of the role of miRNAs potentially involved in angiogenesis control in preterm newborns.

## 1. Introduction

Preterm birth rates range from 7% to 18% worldwide [1]. Despite considerable efforts, the incidence of preterm birth is still rising. Especially, the population of infants born preterm has risen abruptly in the last decades because contemporary perinatal care often reaches 95% of survival rate [1]. At the time of preterm labor, many key organs are structurally and functionally immature. As a result, preterm birth leads to functional adaptations in organs and systems of the newborn that facilitate its survival but may simultaneously increase the vulnerability to multiple organ dysfunctions [2,3].

Multiple reports have defined a distinct cardiovascular phenotype of subjects born preterm, including altered cardiovascular structure and function [4], reduced exercise capacity [5], as well as increased risk of stroke and the development of other circulatory disorders [6]. Recently, preterm birth has been announced as a novel risk factor for heart failure in young people [7], highlighting the importance of experimental and human studies investigating the underlying pathophysiological mechanisms responsible for such increased cardiovascular disease risk in this selected population. Long-term health consequences of preterm birth are gradually being revealed and recent studies of adolescents and young adults reported a higher blood pressure (BP), increased incidence of arterial or pulmonary hypertension, and indices of vascular dysfunction, as well as significant cardiac alterations [8]. Studies from around the world steadily show a dose–response relationship between degree of prematurity and increase in BP. This finally results in a greater risk from stroke mortality and morbidity from other vascular disorders [9,10]. Moreover, it was observed that in women born as preterm, the further pregnancy complications are more common, with a 50% increase in the risk of gestational hypertension, preeclampsia, and arterial hypertension [11]. Likewise, Kajantie et al. reported that preterm women born before 34 weeks, compared to term, have around a 2-fold increase in coronary heart disease as observed in a cohort study of people born in Helsinki [12]. The results from these studies indicate that preterm birth, like poor fetal growth with birth at term, may also program an increase in later cardiovascular risk. The mechanisms behind these malfunctions in subjects born preterm are mostly unclear, but may in part be explained by interrupted cardiovascular system development and maturation occurring during fetal growth and resulting in deficits in vascular regulation in postnatal life.

On the other side, preterm birth is often associated with maternal and neonatal acute distress. Thus, vulnerability of the preterm newborn to stressful intrauterine and extrauterine environmental conditions suggests that preterm labor-related mechanisms may inseparably contribute to increased blood pressure observed in individuals born preterm. Indeed, the reports exist that indicate a key role for the level of maturity of vasculature, the kidneys’ development, and neuroendocrine system function, including abnormalities in the renin-angiotensin system, in pathogenesis of vascular complications and hypertension in subjects born preterm [13,14]. The diminution in the microvascular density and reduced capacity of capillary sprouting are major determinants of increased vascular resistance, thus resulting in the progress of hypertension [15]. Mechanisms linking high BP with capillary malfunction involve endothelial-derived oxidative stress [16], enhanced anti-angiogenic activation [17], and premature vascular aging, resulting in its impaired proliferative and regenerative capacity. These factors would directly impact on capillary sprouting through diminishing the stimulation of pro-angiogenic factors by resident endothelial cells and of endothelial progenitor cells’ (EPCs) recruitment to local neovascularization [18]. Our group has previously found higher early- and late-EPC counts in umbilical cord blood (UCB) of premature infants compared with full-term neonates [19]. Furthermore, we also reported that elevated numbers of early endothelial progenitor cells in the peripheral blood of preterm neonates were associated with retinal injury [20]. Machalinska et al. has also found that in preterm neonates with hypoxic retinopathy, elevated VEGF and IGF-1 blood levels correlated with retinal abnormalities, indicating that these trophic factors may be predictive of abnormal neurodevelopmental outcomes, including retina [21]. Importantly, despite the fact that preterm neonates have higher numbers of EPCs in UCB than full-term infants, these cells, especially the EPC subtype with high proliferative and angiogenic properties, known as endothelial colony-forming cells, were found to have dysfunctional angiostatic properties, when tested in vitro [22]. Vassallo et al. revealed that the accelerated senescence of cord blood EPCs in premature neonates is driven by decreased expression of SIRT1 protein [23]. Moreover, in the studies of Bertagnolli et al., the preterm EPCs population was more vulnerable to exogenous factors, such as oxidative stress, compared to full-term infants [24]. 

MicroRNAs (miRNA) are noncoding RNAs that control diverse biological processes, modulating gene expression at the posttranscriptional level. Each miRNA can individually regulate up to several hundred genes, and they may jointly regulate as much as 50–60% of the transcriptome, suggesting that miRNAs may have pleiotropic effects in the human body [25]. The important functions of miRNAs have been recognized in various biological processes associated with cardiovascular system homeostasis, including angiogenesis and vasculogenesis or endothelial cell proliferation and metabolism [26]. Accumulating studies have suggested that a number of miRNAs are abnormally expressed and stably exist in the blood or its cellular components (e.g., peripheral blood mononuclear cells (PBMCs)), making them ideal circulating biomarkers for early detection in different clinical states. Indeed, our previous analysis of miRNA expression profiling in vascular disorders, including retinal neovascularization, showed a close correlation with clinical symptoms, suggesting that biological activity of circulating PBMCs may reflect the actual state of microvasculature [27,28]. The role of miRNAs has been also reported in the context of neonatal diagnostics and the first description of miRNA profiling in UCB was reported in 2015 [29]. Recent studies have clearly shown that pathologies developing as a result of ischemia or asphyxia may alter the specific miRNA levels in UCB, suggesting their potential role in the early detection of such disorders [29,30]. PBMCs circulating in UCB might be a suitable alternative and valuable source of molecular biomarkers for neonatal diagnostics, thanks to the noninvasive and painless UCB collection at the time of child delivery. However, there are currently no reports demonstrating the use of the level of microRNA expression from UCB as specific biomarkers for any neonatal condition. Additionally, it still remains unclear which miRNAs could be associated with physiological, as well as pathological, conditions of preterm gestation, and which miRNA should form the panel of diagnostic markers for early detection of the cardiovascular system prematurity at birth.

Here, we sought to investigate the levels of several angiopoietic factors in umbilical cord blood from neonates born prematurely, who were otherwise apparently healthy and free of cardiovascular or respiratory diseases and other prematurity-related complications and compare them to term newborns. In addition, using miRNA microarrays and bioinformatics software, we also examined array-based miRNA profiles in UCB-derived PBMCs from the same set of children, comparing preterm and term subjects to identify candidate miRNAs that may contribute to the pathomechanism of this specific clinical condition of the neonates, such as preterm birth and prematurity of the whole cardiovascular system.

## 2. Results

### 2.1. Characteristics of the Study Groups 

We enrolled 27 preterm and 52 term infants in this study. The sociodemographic and medical characteristics of all the examined newborns are summarized in Table 1. The preterm and term neonates differed in their physical measurements, arterial blood gas test results, and several blood laboratory test results or consecutive Apgar scores during the labor. 

### 2.2. Umbilical Cord Blood (UCB) Plasma Cytokines Levels

**Figure 1:**
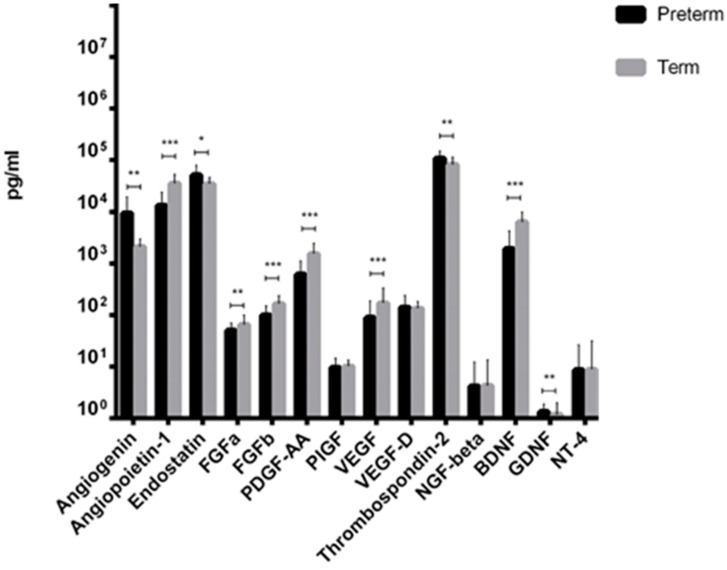
Cytokines levels in Umbilical Cord Blood (UCB) from preterm and term infants (* *p*-value < 0.05; ** *p*-value < 0.01; *** *p*-value < 0.001, Mann–Whitney test).

From fourteen factors tested, ten showed significantly different levels between preterm and term neonates. We observed that Angiogenin, Endostatin, Thrombospondin-2, and GDNF levels were significantly increased in preterm neonates compared to term infants (Figure 1). In contrast, mean concentration of VEGF-A, PDGF-AA, Angiopoietin-1, FGFa, FGFb, and BDNF were significantly decreased in preterm neonates, compared to term infants (Figure 1). Additionally, in the case of two angiogenesis-related molecules, such as PlGF and VEGF-D, and two neurotrophic factors, i.e., NGF, and NT-4, the concentration measured in UCB from preterm and term newborns was almost the same with no statistical difference.

### 2.3. MiRNA Transcripts Expression Profile

**Figure 2:**
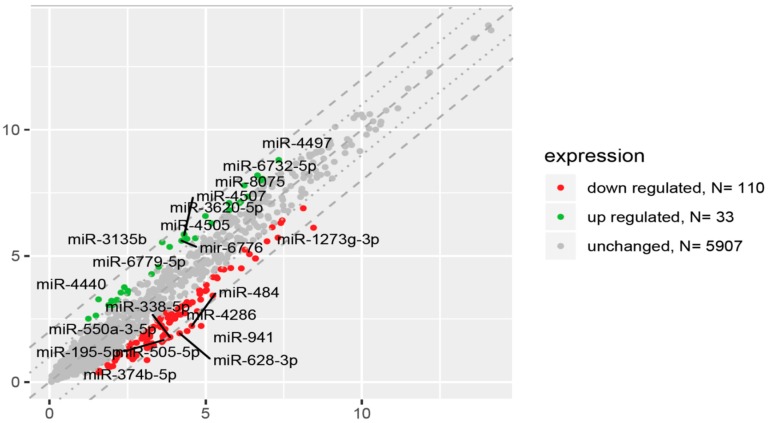
The scatter plot of global miRNA expression in UCB from preterm neonates when compared to term ones. Red points show downregulated miRNAs (at least 2-fold change, *p* < 0.05), green points show upregulated miRNAs (at least 2-fold change, *p* < 0.05). The graphs also comprise the names of miRNAs with the largest change in expression. On the vertical axis (y-axis) are plotted data from preterm group in this study. On the horizontal axis (x-axis) are plotted data from termly delivered neonates, which serve as the control group in this study.

In order to gain an expression profile of miRNAs that is specific to prematurity, the microarray was used to identify the differentially expressed miRNAs in peripheral blood-derived mononuclear cells circulating in UCB obtained from healthy, appropriate-for-gestational-age preterm newborns (*n* = 27) and full-term controls (*n* = 52). We applied a stringent filtering approach that compared healthy preterm newborns with term infants (absolute fold change > 2.0). There were a total of 143 aberrantly expressed miRNAs in preterm newborns compared to term infants that were identified by microRNA array analysis (Figure 2). The significantly differentially expressed miRNAs characterized by high fold change (FC) values ranged for upregulated miRNAs from 2.01 to 3.80 and for downregulated genes from -2.01 to -6.20. All the 33 miRNAs that were significantly upregulated in preterm newborns are listed in Table 2. The highest expression was observed in the case of miR-3135b transcript with a nearly 4-fold difference in the expression compared to term newborns. In an effort to better understand molecular function or biological process difference between miRNA profiles in preterm and term infants, we analyzed the Gene Ontology (GO) of miRNAs target genes. In the group of the significantly upregulated miRNAs, the GO analysis identified the involvement of these miRNAs in various biological processes such as cell cycle (miR-3620), response to hypoxia (miR-3620), DNA repair and recombination (miR-3620), glycerophospholipid biosynthesis (miR-3620), metabolism and metabolism of proteins (miR-3620), Angiopoietin-Like Protein 8 regulatory pathway (miR-3620), process of glucose metabolism (miR-8075), positive regulation of cardiac muscle cell apoptotic process (miR-320), regulation of cell migration involved in sprouting angiogenesis (miR-320), negative regulation of monooxygenase activity (miR-378), positive regulation of angiogenesis (miR-378), regulation of cardiomyocyte hypertrophy (miR-130b), negative regulation of monooxygenase activity (miR-130b), and negative regulation of cholesterol efflux and lipoprotein transport (miR-130b). 

On the other hand, in the group of the significantly downregulated 110 miRNA transcripts in preterm newborns, the GO analysis showed that the mostly down-regulated miR-941 transcript (approximately 6 folds) can participate in the regulation of mitogen-activated protein kinase (MAPK) protein signaling and hormonal signaling of insulin. The other highest downregulated miR-484 transcript (around 5 folds) is involved in the cell cycle and mitosis regulation, organelle biogenesis, and maintenance, as well as signaling provided by GTPases and GPCR molecules. Additionally, highly downregulated miR-338-5p also plays a role in negative regulation of gene expression with gene silencing by miRNA and in the negative regulation of cell migration. The miR-363-3p is involved in Toll-like receptor signaling and actin cytoskeleton regulation. Among the significantly downregulated miRNAs in the preterm newborns, the specific “Angio-MiRs” were found, which have control over the angiopoietic factors. The miR-17-92 cluster regulates the process of positive regulation of vascular smooth muscle cell proliferation, positive regulation of pulmonary blood vessel remodeling, positive regulation of cardiac muscle hypertrophy in response to stress and negative regulation of systemic arterial blood pressure, as well as cellular response to hypoxia and positive regulation of hydrogen peroxide-mediated programmed cell death. miR-93 is involved in DNA damage response. miR-125 controls the process of positive regulation of endothelial cell apoptotic process, positive regulation of sprouting angiogenesis, and negative regulation of glomerular mesangial cell proliferation, as well as negative regulation of angiogenesis. miR-126 controls positive regulation of blood vessel endothelial cell migration, positive regulation of MAPK cascade, positive regulation of ERK1 and ERK2 cascade, positive regulation of Notch signaling pathway, cellular response to hypoxia, negative regulation of endothelial cell apoptotic process, positive regulation of vascular endothelial cell proliferation, positive regulation of vasculature development, and positive regulation of blood vessel endothelial cell proliferation involved in sprouting angiogenesis. miR-145 regulates vascular smooth muscle cell differentiation, aorta smooth muscle tissue morphogenesis, positive regulation of cardiac vascular smooth muscle cell differentiation, negative regulation of cardiac muscle cell apoptotic process, and myofibroblast differentiation with angiotensin-activated signaling pathway and negative regulation of angiogenesis. miR-150 controls positive regulation of endothelial cell differentiation, positive regulation of cell migration involved in sprouting angiogenesis, positive regulation of mesodermal cell differentiation. miR-155 controls negative regulation of vascular wound healing, negative regulation of cell migration involved in sprouting angiogenesis, positive regulation of cardiac muscle hypertrophy in response to stress, negative regulation of blood vessel endothelial cell proliferation involved in sprouting angiogenesis, negative regulation of leukocyte adhesion to vascular endothelial cell, regulation of apoptotic cell clearance, positive regulation of reactive oxygen species biosynthetic process. The miR-210 is involved in regulation of cellular response to hypoxia, hypoxia-inducible factor-1 alpha signaling pathway and positive regulation of apoptotic signaling pathway, as well as in the positive regulation of angiogenesis and tube formation, positive regulation of cell migration and positive regulation of blood vessel endothelial cell migration, and negative regulation of vascular associated smooth muscle cell apoptotic process.

**Figure 3:**
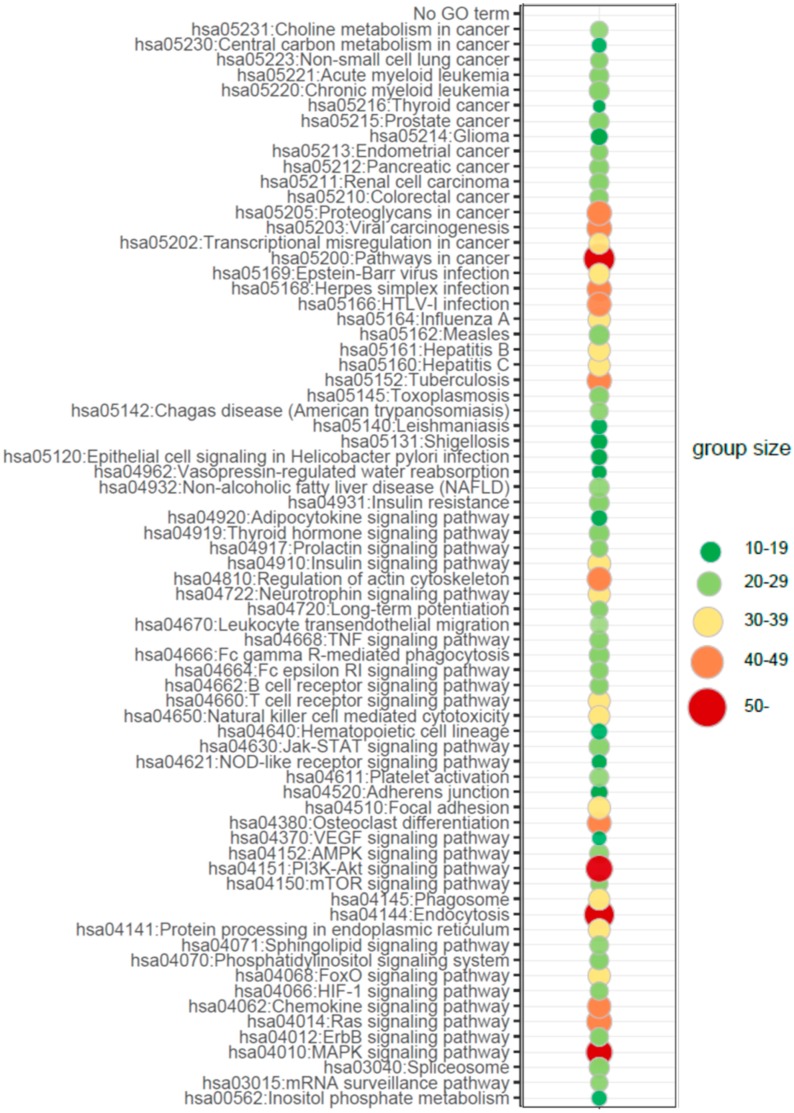
Bubble plot of overrepresented gene sets in DAVID Gene Ontology-Biological Process (GO BP) annotations database obtained from comparisons in miRNA transcript expression profiles between preterm vs. term newborns. The graph displays only the Gene Ontology (GO) groups above the established cut-off criteria (*p* with correction < 0.05, a minimal number of genes per group > 10). The color of each bubble, according to the legend, reflects the number of differentially expressed genes, assigned to the GO BP terms. Interestingly, analysis of biological processes regulated by differentially expressed miRNAs target genes revealed processes of characteristic for the regulation of angiogenesis and activity of endothelial cells.

To determine which biological processes can be differentially regulated by microRNAs globally in newborns from preterm labor, we performed the additional analysis of the enrichment in the relevant ontological groups from the Gene Ontology-Biological Process (GO BP) direct database. Thus, miRNA targets were assigned to (GO BP) database. A whole set of differentially expressed microRNA transcripts consisting of 143 miRNAs (33 up- and 110 down-regulated) was subjected to functional annotation and clusterization using the database for annotation, visualization, and integrated discovery (DAVID) bioinformatics tools. The result of this analysis is displayed as a bubble plot (Figure 3), which shows only ontological groups fulfilling the following criteria: adjusted *p*-values below 0.05 and minimal number of genes per group =5. According to GO analysis in PBMCs circulating in UCB from preterm newborns, there are nearly one hundred significantly differently expressed miRNA-target pairs playing an important role in the regulation of signaling pathways and endothelial cells activities, inter alia: “VEGF signaling pathway (04370)”, “Neurotrophin signaling pathway (04722)”, “MAPK signaling pathway (04010)”, “PI3K-AKT signaling pathway (04151)”, “HIF-1 signaling pathway (04066)”, “Sphingolipid signaling pathway (04071)”, “Chemokine signaling pathway (04066)”, “Leucocyte transendothelial migration (04670)”, “Adherens junction (04520),” or “Endocytosis (04144)”.

**Figure 4:**
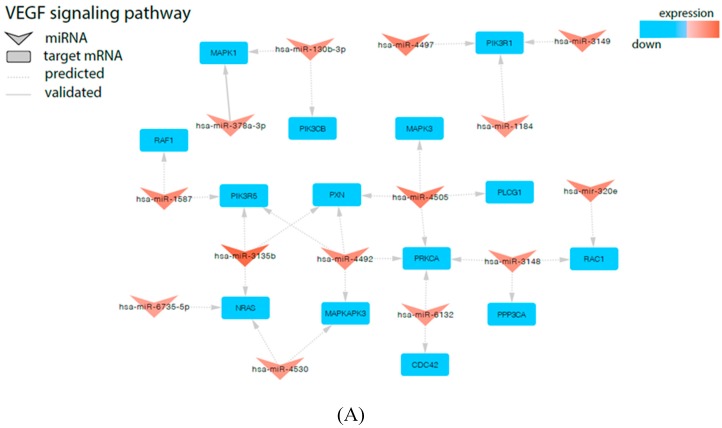

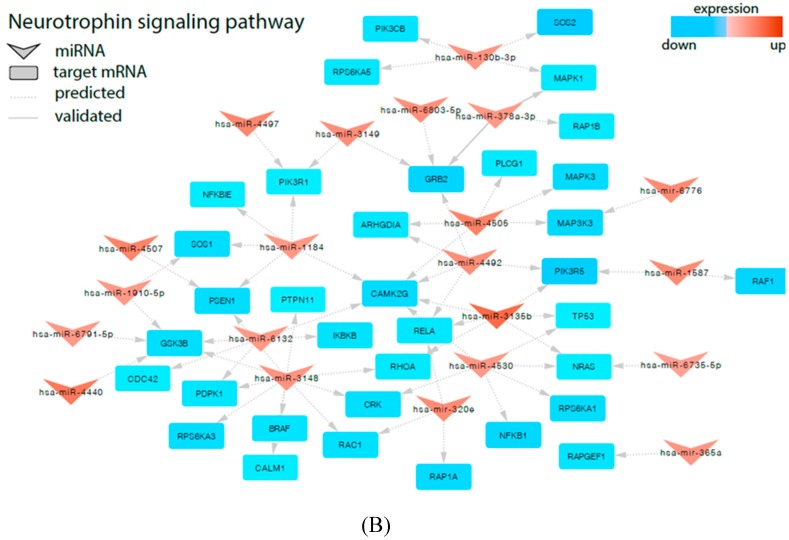
Signaling pathways of Vascular endothelial growth factor (VEGF) (**A**) and Neurotrophins (**B**) involved in regulation of angiogenesis and proliferation/apoptosis are modified in UCB from newborns from preterm labor. The arrowheads represent differentially expressed miRNAs and the squares represent negatively regulated genes.

Next, in the subsequent analysis to check the relationship between miRNAs and genes regulated by them that are specifically involved in the processes, such as proliferation/apoptosis and angiogenesis regulation, we revealed that in PBMNs from UCB of newborns after preterm labor, these genes were controlled by the range of significantly up-regulated miRNAs (Figure 4A): *CDC42* (miR-6132), *MAPK1* (miR-130b-3p, miR-378a-3p), *MAPKAPK3* (miR-4492, miR-4530), *NRAS* (miR-3135b, miR-4530, miR-6735-5p, let-7b-5p, let-7c-5p), *PIK3CB* (miR-130b-3p), *PIK3R1* (miR-1184, miR-3149, miR-4497), *PIK3R5* (miR-3135b, miR-1587, miR-4492), *PLCG1* (miR-4505), *PPP3CA* (miR-3148), *PRKCA* (miR-6132, miR-3148, miR-4492, miR-4505), *PXN* (miR-3135b, miR-4505, miR-4492), *RAC1* (miR-3148, miR-320e), and *RAF1* (miR-1587). It was found that these genes are engaged in several processes taking place during cardiovascular system development (sprouting angiogenesis and vascular endothelial growth factor receptor signaling pathway: *CDC42*, *PIK3CB*; positive regulation of cardiac muscle cell proliferation and cardiac neural crest cell development involved in heart development: *MAPK1*; positive regulation of blood vessel endothelial cell migration: *PLCG1*; heart development: *RAF1*), MAPK cascade regulation (activation of *MAPK* and *MAPKK* activity and regulation of stress-activated MAPK cascade: *MAPK1, MAPKAPK3, NRAS, PIK3CB, RAF1*), neoangiogenesis (positive regulation of endothelial cell proliferation: *NRAS, PIK3CB*; positive regulation of angiogenesis and positive regulation of endothelial cell apoptotic process: *PLCG1*; vascular endothelial growth factor receptor signaling pathway: *PXN, RAC1*), wound healing (angiogenesis involved in wound healing and platelet aggregation and activation: *PIK3CB, RAF1*; response to wounding: *PIK3CB, RAC1*), intrauterine organism development (in utero embryonic development: *PLCG1*; anatomical structure morphogenesis: *RAC1*), and cardiac and vascular function (positive regulation of cardiac muscle hypertrophy and cardiac muscle hypertrophy in response to stress: *PPP3CA*; regulation of nitric oxide biosynthetic process: *RAC1*). 

Similarly, we observed that many of the miRNAs, which are highly upregulated in UCB from preterm newborns, were cross-linked with genes belonging to the Neurotrophins signaling pathway, thus displaying the negative regulation of this pathway (Figure 4B). The overexpressed miRNAs specifically regulate the expression of following genes: *BRAF* (involved in cellular response to nerve growth factor stimulus and positive regulation of axonogenesis), *PTPN11* (BDNF signaling pathway and neurotrophin TRK receptor signaling pathway), *PSEN1* (regulation of neurite outgrowth and neuron development, neuron apoptotic process and negative regulation of neuron apoptotic process and transmission, as well as in synaptic vesicles replenishment in a process of presynaptic facilitation), *PLCG1* (BDNF signaling pathway and neurotrophin TRKA receptor binding), *RELA* (regulation of Schwann cell differentiation), *TP53* (development of neurotrophin family signaling, regulation of neuron apoptotic process, and regulation of neuroblast proliferation), *GRB2* (Neurotrophin TRKA receptor binding), *RAC1* (neuron migration and motor neuron axon guidance), *RAF1* (Neurotrophin TRK receptor signaling pathway), *CRK* (neuron migration, cerebellar neuron development and neurotrophins signaling pathway). Our finding also demonstrated the involvement of several miRNAs involved in a neurotrophin signaling pathway controlling neural growth and proliferation through potential regulation of MAP kinase cascade activity (*MAPK1*, *MAPK3*, *MAP3K3, BRAF, PTPN11, NRAS, RAF1*, and *CRK*). Therefore, we found an extensive regulatory network between miRNAs, which are highly expressed in PBMN cells in UCB and their corresponding genes.

### 2.4. RNA Gene Expression Profile

**Figure 5:**
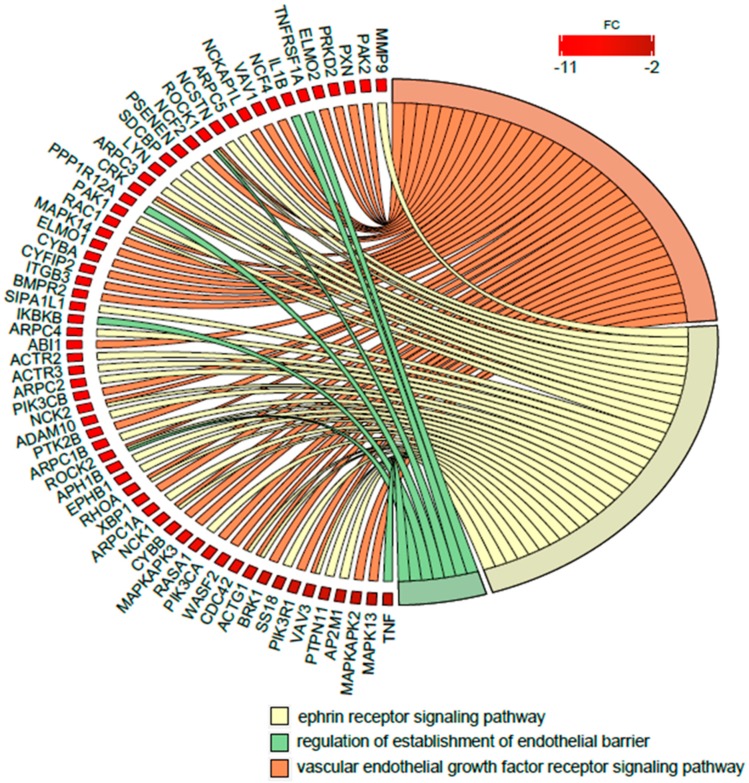
Detailed analysis of three enriched gene ontological groups involved in angiogenesis regulation in preterm newborns. Circos plots with interdependence between selected GO terms and their genes. Symbols of differentially expressed genes (DEGs) are presented on the left side of the graph with their fold change values, mapped by color scale (red = lower expression). Moreover, the color intensity of the rectangle next to the marked gene name reflects the order of magnitude of the change in gene expression. Gene involvement in the GO terms was determined by colored connecting lines.

Finally, because the DAVID BP bioinformatics tool revealed the significant changes of expression of miRNA transcripts strongly related, among others, to regulation of angiogenesis as well as proliferation/apoptosis in newborns from preterm labor, in the next step we performed an analysis of the enrichment in the relevant ontological groups from the DAVID GO BP direct mRNA gene database. A whole set of differentially expressed genes (DEGs) consisting of 4031 genes (250 up- and 3781 down-regulated) was subjected to functional annotation and clusterization, using again the DAVID software. As a result of this specific analysis, the expression of mRNA encoding genes belonging to the following ontological groups were found: (i) Vascular endothelial growth factor receptor signaling pathway (GO: 0048010), (ii) Regulation of establishment of endothelial barrier (GO: 1903140), and (iii) Ephrin receptor signaling pathway (GO: 0048013). Of note, the latter one encompasses the signaling of Ephrin receptors, a family of receptor tyrosine kinases, which are crucial for vascular endothelial cell migration and adhesion during angiogenesis and neovascularization, thus playing an important role in vascular and neuronal development, as well as functioning as activators of the MAP kinase cascade. Due to the ambiguous nature of the Gene Ontology structure, single genes can often be assigned to many ontological terms. Therefore, the relationship between genes and GO terms were mapped with Circos plots with visualization of logFC values and gene symbols (Figure 5). The strongest down-regulated genes from the examined ontological groups included inter alia: *MMP9*—Matrix Metallopeptidase 9 (involved in neovascularization as a key regulator of angiogenesis), *PAK2*—*P21* (*RAC1*) Activated Kinase 2 (involved in Angiopoietin-TIE2 Signaling and MAPK signaling pathway), *PXN*—Paxillin (involved in Angiopoietin receptor Tie2-mediated Signaling and VEGF—VEGF-R2 Signaling), *PRKD2*—Protein Kinase D2 (plays a regulatory role in angiogenesis by phosphorylating the downstream effectors resulting in VEGF secretion), and several MAP kinases (MAPK13, MAPK14, MAPKAPK2, MAPKAPK3) involved in many cellular processes, including gene expression regulation and cell proliferation, together with angiogenesis and neovascularization.

## 3. Discussion

There are only a few previous studies aimed at identifying cord blood biomarkers of angiogenesis and the vascular compartment in preterm newborns. Here, for the first time, our group has performed the complex analysis of several angiopoietic factors and other trophic factors that drive vasculature development and endothelial homeostasis in newborns with a low gestational age that were born prematurely. We found that several pro-angiogenic factors, including Angiopoietin-1, VEGF, FGF-acidic, FGF-basic, PDGF-AA, and BDNF, were significantly decreased in the UCB of premature infants. In contrast, the UCB concentration of crucial angiostatic molecules, such as Endostatin, and Thrombospondin-2, was significantly increased in this group. Additionally, two other factors which support angiogenesis, i.e., Angiogenin and GDNF, were significantly upregulated in UCB of preterms. These findings contribute to our growing understanding of the role of fetal angiogenesis and endothelial homeostasis in the pathophysiology of premature birth and prematurity itself. 

Angiogenesis, together with vascular growth (vasculogenesis) during fetal life, is dependent on endothelial cells, numerous growth factors, and cytokines, of which angiopoietic factors seem to be very important [31]. Vascular growth is driven by vascular endothelial cells, forming stable connections and cellular rearrangements during sprouting, anastomoses, lumen formation, and functional remodeling of the vascular network [32]. The biological state of vascular endothelial cells is an important regulatory factor for the homeostasis and quality of vasculature development in preterm newborns. Intrauterine growth of the vascular bed during gestation is regulated by a number of trophic factors, particularly VEGF, which is an endothelial cell-specific regulator of vascular permeability and angiogenesis, which facilitates sprouting and proliferation of endothelial cells [33]. In endothelial cells, VEGF induces expression of cell-adhesion molecules and the release of other trophic factors and cytokines [34]. The pro-angiogenic Angiogenin promotes endothelial cell activities, including migration, proliferation, and tube formation by interacting with endothelial cells [35]. Several fibroblast growth factors induce migration of endothelial cells and reorganization of the tube structure [36]. Placental growth factor, a related homolog of VEGF, also induces angiogenesis [37]. Likewise, platelet-derived growth factor modulates endothelial proliferation and angiogenesis via its receptor on endothelial cells [38]. In addition, the local oxygen environment plays an important role in development and control of organ vasculature. The angiopoietic growth factors have been implicated in various vascular diseases, including atherosclerosis, but little is known about their expression and role in angiogenesis regulation and vascular development in premature fetuses and the postnatal life of preterm newborns.

Literature data indicate that angiogenin (ANG) is a pro-angiogenic factor essential for cell proliferation and angiogenesis induced by other angiogenic factors [35]. In particular, it has been reported that nuclear translocation of ANG is a critical step for angiogenesis induced by other growth factors including VEGF, FGF, or EGF [39]. ANG was also shown to trigger nitric oxide synthase (NOS) activity in human umbilical vein endothelial cells (HUVECs), inducing increased vascular permeability and vasodilatation [40]. Previously, it was reported that fetal circulating ANG levels did not differ between pregnancies with small-for-gestational-age fetuses and appropriate-for-gestational-age fetuses, thus it might indicate that only pathophysiological processes during preterm gestation could affect ANG production and its levels in peripheral blood, but not the body size of the fetus [41]. Another study reported that serum ANG levels were higher in pregnancies complicated by hypertension than in normal, healthy pregnancies [42]. Here, we showed that from several pro-angiogenic factors tested in this study, only ANG appeared to be significantly increased in UCB from preterm newborns. Our observation of higher levels of ANG in preterm newborns may indicate its potential role in vascular development during preterm gestation.

On the contrary, we observed decreased levels of several pro-angiogenic factors in UCB from preterm babies. VEGF is expressed in villous and extravillous trophoblasts and its expression level alters with adverse pregnancy outcomes [43]. It has been observed that pregnancies with placenta that demonstrated severe vascular under-perfusion were strongly associated with lower levels of proangiogenic factors in UCB, such as VEGF and its soluble receptor sVEGFR-2 [44]. It was found that resident placental endothelial cells from pregnancies characterized by fetal growth restriction resulting in preterm delivery, demonstrated decreased angiogenesis with impaired signaling in different VEGF pathways, including activation of the VEGF–NO signaling [45]. Importantly, previously published data indicated that VEGF levels were significantly lower in UCB from premature fetuses than in term fetuses [46]. Moreover, the concentration of numerous pro-angiogenic factors was diminished not only in UCB but also in the peripheral blood collected from pregnant women, who delivered preterm neonates. For example, plasma concentration of PlGF and sVEGFR-2 were lower in pregnant women even at 5 weeks prior to the diagnosis of spontaneous preterm delivery [47]. In this notion, we analyzed a large number of pro-angiogenic molecules in UCB samples from preterm newborns and confirmed the general profile of UCB with decreased concentration of several pro-angiogenic factors in neonates born prematurely. The reduction of systemic FGF-basic observed in our study is particularly interesting, since this factor has been shown to act in cooperation with VEGF to accelerate vascularization.

The role of Angiopoietin-1 in fetal cardiovascular development is not well understood. Low levels circulating at preterm birth may represent a primary defect in production by the dysfunctional endothelium and circulating endothelial progenitor cells, impaired upstream regulators, or competition with Angiopoietin-2 for the common receptor Tie-2 in peripheral premature vasculature [48]. Existing data suggest that both Angiopoietin-1 and VEGF act in a very complementary and coordinated fashion, with former molecules to promote vessel branching and remodeling, as well as to promote maturation and stabilization of vessels [49]. Critical to the function of Angiopoietin-1 appears to be its ability to optimize interactions of endothelium with surrounding supporting cells and matrix, thus stabilizing and maintaining vessels [45]. The defects in mice lacking Angiopoietin-1 suggest that this protein is critical for normal remodeling, maturation, and stabilization of the developing vasculature [50]. Additionally, the microscopic examination of vessels in animals lacking Angiopoietin-1 showed that endothelial cells failed to interact and adhere properly and vessels failed to follow normal remodeling and were at risk for subsequent regression. Another major defect in mice lacking Angiopoietin-1 involves abnormal heart development with several deformities in heart structure, including heart chambers trabeculation and endocardium quality [50]. Here, our findings demonstrated that these angiopoietic factors that biologically cooperate together, VEGF-A and Angiopoietin-1, were also both affected by premature birth, as they were significantly decreased in UCB samples from preterm infants population and could exert abnormal humoral effects on cardiovascular system. Although the decrease in the levels of pro-angiogenic factors in our study might be a result of ongoing angiogenesis-related processes in the developing peripheral organs, with quick turnover of selected angiogenesis-stimulating factors, however, we cannot exclude that the cause of those disturbances could be the prematurity of endothelial cells in microvasculature and that they are the main producers of angiogenic growth factors in the growing organism. This requires further in-depth studies and correlation with Angiopoietin-2 levels that permits the fetal blood vessels to undergo remodeling during gestation through branching and non-branching angiogenesis [51].

During pregnancy, the interactions between oxygen partial pressure, different vascular cell types, and the regulation of cellular responses, such as angiopoietic factors (e.g., VEGF and PlGF, and their receptors), lead to proper control of angiogenesis. Postulated changes in the vasculature development in infants born prematurely may also be estimated by analysis of the ratio of two specific angiogenic proteins, i.e., VEGF/PlGF ratio. PlGF appears to augment the angiogenic effect of VEGF. It was found that specific type of angiogenesis with increased branching that permits for better oxygen and nutrients distribution into peripheral tissues is likely a result of a greater VEGF/PlGF ratio with elevated VEGF concentrations in relation to PlGF [52]. In contrast, angiogenic imbalance characterized by decreased VEGF/PlGF ratio may have a causative role in impaired vascularization, especially in placenta, prior to the development of preeclampsia [53]. The analysis of the VEGF/PlGF ratio in our cohorts of preterm and term newborns confirms the imbalance between these two angiogenic factors in the former group. These findings might demonstrate the adverse influence of lower circulating VEGF found in preterm newborns on vasculature control and endothelial homeostasis in these subjects. Likewise, with regard to VEGF/PlGF values in UCB samples from preterm newborns, combining clinical data with oxidative blood status and vascular/angiopoietic biomarkers such as VEGF/PlGF, might potentially be a good ratio index for clinical use for predicting the influence of preterm birth on final outcomes related to prematurity. However, the real impact of VEGF/PlGF ratio requires further large scale studies [54].

Several proteins with anti-angiogenic properties are associated with adverse neonatal outcomes. Some of them have been implicated in the pathophysiology of anti-angiogenic states of pregnancy, including preterm labor [50], small-for-gestational age [55], fetal death [56], twin to twin transfusion syndrome [57], or preeclampsia [58]. This state is a result of the imbalance in the production of angiogenic factors, such as VEGF, and different anti-angiogenic factors. It is likely that the elevation in concentration of potent anti-angiogenic factors in infants with preterm labor is not a signal to initiate such labor, but rather reflects longitudinal general perturbations in preterm gestation [47]. In this notion, we have detected increased concentration of two independent and potent angiostatic factors, i.e., Endostatin and Trombospondin-2 in UCB from preterm infants compared to term newborns. Consistent with our findings, Janer et al. detected a high concentration of Endostatin in UCB from very low birth weight infants [59]. Interestingly, Lewandowski et al. has observed enhanced anti-angiogenic state even in young adults who were born as preterm babies, characterized by increased endoglin and soluble sVEGFR-1, which both are the potent anti-angiogenic factors [17]. Besides, in the in vivo model, the intraamniotic administration of anti-angiogenic sVEGFR-1 to animals in preterm gestation could decrease pulmonary vessel density, suppress activation of VEGF receptor-2, and increase apoptosis in endothelial cells in the newborn lungs, suggesting that an elevation of anti-angiogenic factors in the preterm gestation may result in impaired vascular growth and reduction of the pulmonary alveolar numbers. Collectively, the results of previous studies coupled with our findings indicate that it is possible that the increased production of potent anti-angiogenic factors may be associated with subsequent development of apoptosis and malfunction in different components of the growing vasculature, especially in endothelial cells. These cells naturally secrete plenty of angiogenesis-regulatory molecules, and probably the diminished population of such cells with reduced functionality in preterm gestation could be one of the causes of impaired concentration of several angiopoietic factors examined in our study in UCB from preterm neonates.

Neurotrophins (NTs) promote survival and reduce apoptosis in many cell populations and their action is not limited to nervous tissue, as their role in angiogenesis has also been proposed [60]. Abrupt removal of the maternal passage and their placental sources can cause the reduction in NTs levels at birth. Because NTs might also play important roles as regenerative factors in cardiovascular system [61] and promote angiogenesis, we decided to compare changes in expression levels of selected classic neurotrophins, including BDNF, GDNF, NGF, and NT-4 in UCB from preterm and term babies. The data presented here demonstrated that preterm birth affects the expression of several neurotrophic factors in UCB. We have shown that the secretion of BDNF was significantly decreased in UCB after preterm labor. BDNF is essential for neuronal development and thus it can be treated as marker of neuronal maturation. Decreased BDNF levels in preterm newborns may be a manifestation of immaturity of neuronal cell population throughout the whole body [62]. Low levels of BDNF in UCB may also indicate a higher risk of neurodevelopmental complications in postnatal life [63]. Similar effects of preterm birth on NTs levels were previously observed by other groups. Matoba et al. reported that BDNF and NT-3 levels were significantly decreased in UCB from preterm birth [64]. BDNF and NT-3 are also involved in the regulation of angiogenesis. Recent studies suggest that the levels of BDNF and other NTs are regulated by docosahexaenoic acid (DHA) which is an important omega-3 fatty acid. Oxidative stress developing in the pregnancy complicated with preterm birth may lower the levels of DHA, thus leading to the observed changes in the levels of BDNF [65]. In this notion, decreased expression of BDNF in the preterm gestation might lead to abnormal fetal growth that may increase the risk of cardiovascular diseases and metabolic abnormalities in the postnatal life of children born preterm.

In contrast, our study demonstrates that the secretion of another important neurotrophic factor, GDNF, was significantly increased in UCB under stress-related conditions during the preterm labor. Similar to our results, Rajkumar et al. observed that cord blood GDNF levels were significantly higher in preterm newborns compared to term newborns [66]. GDNF is a potent neurotrophin that protects the central and peripheral nervous system against degeneration. Previous experimental studies have shown the protective effect of GDNF in enteric neuronal survival, which seems to cooperate with classical NTs in the pathological and physiological states of gut development [67]. Here, increased levels of GDNF during preterm birth may represent a compensatory mechanism to protect preterm newborns in the course of reduced production of other classical NTs and other trophic factors, such as VEGF. Interestingly, there are existing crosstalks between GDNF or its putative receptors with the Semaphorins/VEGF family [68]. Thus, GDNF may play an important role in vascular development during preterm gestation.

Exploring the effects of preterm birth on microRNAs expression in UCB, the microarray analysis carried out in our laboratory showed that preterm gestation led to differential expression of 143 miRNAs, which were either significantly down- or up-regulated. Further bioinformatic analysis revealed that up-regulated 33 miRNA sequences may be also engaged in several biological processes, which may regulate growth, survival, and differentiation of endothelial and other vascular-related cells. Endothelial function is partially manifested by the permeability and migration. Indeed, the highly upregulated miRNA miR-4505 from our study is associated with control of the transcription of heat shock protein family members, also those primarily expressed in endothelial cells to participate in vascular network formation and cell migration. It was reported that the expression level of miR‑4505 increases after LPS stimulation in endothelial cells from umbilical cord and this mechanism may involve NF‑κB activation. As a result, miR‑4505 induces high permeability and reduces migration of endothelial cells [69], demonstrating that miR‑4505 may aggravate endothelial impairment in the vasculature of preterm newborns. It might be speculated that increased expression of miR-4505 is one of the intrinsic feedback mechanisms involved in endothelial injury during acute stress in course of preterm birth. In our study, another upregulated miRNA involved in the regulation of cellular functions, including cell proliferation and apoptosis, was miR-4530. It has been revealed that overexpression of miR‑4530 can suppress cell proliferation and enhance apoptosis in human umbilical vein endothelial cells (HUVECs) via the ERK/MAPK and PI3K/AKT signaling pathways [70]. Furthermore, it was reported that miR‑4530 was upregulated in the blood of patients with diabetic retinopathy [71]. Recent analysis published by Chen et al. showed that miR-4530 might be involved in the development and progression of unexplained recurrent spontaneous abortions [72]. It was also established that miR-4530 plausibly contributes to the promotion of angiogenesis with endothelial cells, maintaining the pro-angiogenic function in tumor formation [73]. There is a report indicating that overexpressed miR-7977 could induce disturbances in the expression of several trophic factors in BM stromal cells that are essential for self-renewal of HSCs, including Angiopoietin-1, which usually induces functional and mature vascular formation [74]. In the present study, we observed in our cohort of preterm newborns, the significant up-regulation of miR-7977 in UCB that might result in a decreased angiogenesis-supporting capacity of BM CD34+ cells in preterm gestation through the reduction of angiopoietic factor levels in blood, such as Angiopoietin-1. In the other study, Chen et al. have demonstrated in in vitro experiments that miR-7977 expression might be an important modulator involved in the molecular mechanisms of cell cycle, proliferation, and apoptosis [75]. Hence, miR-7977 could play an important role in the disturbance of fetal angiogenesis. This suggests that deregulation in miRNA expression may be crucial in the pathogenesis of prematurity-related angiopoietic factors misbalance (angiogenic vs. angiostatic) via regulation of selected miRNA biogenesis. Although the precise function of the overexpressed miRNAs in the preterm fetal organism remains unknown, these miRNAs seem to be good candidates for further assessment, as diagnostic biomarkers preceding the clinical onset of pathologic states in the postnatal life that are nowadays related to the prematurity.

We further performed functional bioinformatics analyses with regard to significantly down-regulated miRNAs in UCB from preterm infants. Although for some of these miRNAs the relationship to angiogenesis-regulating functions was mostly unknown, among them there were also extensively studied molecules called “Angio-MiRs”, such as *miR-125*, *miR-126*, *miR-145*, *miR-150*, and *miR-155*, which demonstrated their participation in various biological processes related to physiological and pathological functions in the cardiovascular system, including cardiovascular system development, different vascular endothelial growth factor receptor signaling pathways, mitogen-activated protein kinase (MAPK) signaling cascade, calcium ion signaling, endothelial cell receptor interactions, angiopoietic factors production, cytokine expression and vascular inflammation, etc. [76]. Among these, inflammation, cell adhesion, T-cell proliferation, calcium transfer, and apoptosis are closely related to the pathological process of endothelial inflammation and atherogenesis [77]. In this notion, vascular-related miR-125a and miR-126 have been shown to be key, pleiotropic promotors of endothelial health and vasomotor function. Furthermore, altered circulating levels of both miRNAs have been shown to be indicative of elevated vascular inflammation and endothelial dysfunction and predictive of cardiovascular morbidity and mortality [78]. For example, miR-126 has shown in different studies the greatest dependency on platelets and is strongly correlated with plasma levels of P-selectin, platelet factor 4, and platelet basic protein [79]. MiR-126 affects also ADAM9 protein, which regulates the motility of cells via interactions with integrins as well as releases a number of molecules with important roles in angiogenesis, such as KDR and VCAM-1. Significantly, down-regulation of miR-126 could reduce the platelet aggregation in the mice model [80]. Conversely, miR-145 and miR-150 limit immune cell activation, cytokine production, and vascular inflammation [80,81]. Our observation of lower expression levels of the above-mentioned angiogenesis-specific subset of miRNAs, which have been identified as crucial regulators of vascular biology and endothelial cell function, may indicate their potential angiogenesis-regulating role in premature gestation coupled with angiopoietic proteins imbalance in UCB found in this study. In this notion, the recent bioinformatics analyses have shown that miR-941, which appeared to be the most down-regulated microRNA in preterm newborns in our study, may be associated with metabolism, inflammation, cell proliferation, and other biological processes through regulation of components involved in insulin signaling, MAPK signaling cascade, and other pathways related pathophysiologically to coronary heart disease and atherosclerosis [82]. However, no research has yet been reported on the role of miR-941 in angiogenesis in course of preterm gestation. What is more, down-regulation of several “Angio-miRs” could be observed in different types of vascular disorders in adult patients. For example, our recent analyses showed that in the blood of patients with age-related retinal degeneration, which is characterized by abnormal vascularization and subsequent macular dysfunction, several significantly down-regulated miRNAs were detected, such as miR-17, miR-21-3p, miR-150, and miR-155, and they were associated with lower blood levels of FGF-basic molecule with potential angiogenesis-regulating function [27,28]. Similar results were obtained from computational analysis of miRNA expression in UCB from prematurely-born infants in this study. This could be interpreted as an indirect evidence for the involvement of selected “Angio-miRs” in microvasculature function in adults and in its development during the physiologic gestation.

In summary, one of the most striking findings in this study was the downregulation in UCB from preterm infants of several miRNAs related to the angiogenic microenvironment that is comprised of many cell types, such as endothelial cells, macrophages, smooth muscle cells, fibroblasts, pericytes, adipocytes, and their trophic products (growth factors, cytokines, chemokines, and extracellular matrix). The bioinformatics functional predictions showed in our study that several differentially expressed miRNAs in the cohort of preterm newborns could be related to the pathological processes in the cardiovascular system, implicating the potential development of different vascular-related diseases in the postnatal life. These observations especially support the suggestion that several up- and down-regulated miRNAs detected by our group in UCB may participate in the cardiovascular-related stress, which could contribute in part to the etiology of preterm birth during the physiologic gestation. Detailed bioinformatics analyses of these miRNAs should be additionally performed and the molecular mechanisms, by which these miRNAs participate in the pathogenesis and onset of preterm birth, must be further explored. 

## 4. Conclusions

Our data elucidates a unique profile of several angiopoietic factors in UCB of preterm neonates. These results may suggest that the analyzed circulating angiopoietic factors found to be abnormally secreted to the newborn’s UCB may contribute to the angiogenesis-related pathological processes observed in clinical practice with children and adults born prematurely. Moreover, our data demonstrated significant changes in the levels of 143 transcripts of miRNA in PBMCs circulating in UCB from preterm newborns, suggesting that these miRNAs are potentially involved in the pathogenesis of preterm birth. However, this study has several limitations. It is not known how these changed expression levels might relate to the actual expression levels within tissues, particularly in the cardiovascular system, including vessels and myocardium. Examination of the peripheral tissues would help to better understand the mechanism of the altered expression and its biological effects. Future research should detect the potential downstream targets of miRNAs detected here to evaluate whether they play an active role in the pathophysiology of preterm gestation. Moreover, expanding the use of these aberrant miRNAs profiles as potential biomarkers to the field of neonatology might shed a light on the early prediction of preterm infants with poor cardiovascular outcomes. Likewise, further studies are also needed to assess how angiopoietic factor levels vary over time, and whether the imbalance in angiogenic and angiostatic factors contributes to the development of different prematurity-related vascular diseases. Future studies are also warranted to determine the mechanistic links between antenatal angiopoietic factors with disease pathogenesis and how these factors impact cardiovascular outcomes that are essential for further advances that may lead to establishing primary prevention of the late abnormal vascular sequelae of prematurity.

## 5. Methods

### 5.1. Characteristics of the Study Group

We enrolled 27 preterm infants born at less than 37 weeks gestational age (GA) (33.0 ± 2.5) and 52 term infants born at >37 weeks GA (38.4 ± 1.0) in the Department of Obstetrics and Gynecology of the Pomeranian Medical University in Szczecin, Poland. All recruited subjects were born by caesarean delivery and were appropriate-for-gestational-age. Exclusion criteria were: multiple gestation births, major congenital malformations, or known chromosomal abnormalities, cyanotic heart defects, intracranial hemorrhage, states of chronic intra-uterine hypoxia (defined as foetal growth restriction or pathologies of placental perfusion), inherited metabolic disorders, congenital infections and severe infectious diseases, maternal history of tobacco and alcohol abuse, severe anemia, with hemoglobin < 120 g/L, and missing parental consent. Only infants who survived to hospital discharge were included. Each child’s sex, GA, birth weight, Apgar score, and clinical course were documented. We adhered to the tenets of the Declaration of Helsinki, and approval was obtained from the Local Research Ethics Committee on 25th January of 2010. Parents provided written informed consent for their children.

### 5.2. Umbilical Cord Blood Collection

Autologous umbilical cord blood (UCB) was collected at birth in accordance with NetCord-FACT international standards for cord blood collection [83]. Collections were performed in utero into a collection bag system with citrate-phosphate-dextrose anticoagulant solution [84]. UCB samples were then centrifuged in a tabletop refrigerated centrifuge (2000 rpm, 4 °C, 10 min), and the plasma was removed from the cell pellet and stored at −20 °C to −80 °C until assayed. In the cell pellet, the red blood cells were lysed using BD Pharm Lyse lysing buffer (BD Biosciences, San Jose, CA, United States) for 15 min at room temperature to isolate peripheral blood mononuclear cells (PBMCs).

### 5.3. Luminex Assay 

Simultaneous measurement of 14 biomarkers, including Angiogenin (ANG), Angiopoietin-1, Endostatin, Fibroblast growth factors (FGF): FGF-acidic, FGF-basic, Platelet-derived growth factor (PDGF-AA), Placental growth factor (PlGF), Vascular endothelial growth factor (VEGF), VEGF-D, Thrombospondin-2, BDNF, GDNF, NGF, and NT-4 was performed. Their concentrations were measured in UCB plasma by multiplex fluorescent bead-based immunoassays (Luminex Corporation, Austin, TX, USA) using commercial R&D Systems Human Angiogenesis A Premixed Mag Luminex Performance Assay (R&D Systems, Minneapolis, MN, USA). 100 μL of blank standards and samples were added to the plate together with a microparticle cocktail and incubated in the dark for 2 h at room temperature on horizontal orbital microplate shaker set at 800rpm. After this step, the wells were washed with 100 µL of wash buffer three times by using a hand-held magnet. A biotin-antibody cocktail (50 µL) was added to the plate and incubated with agitation at room temperature for 60 min in the dark. After washing, 50 µL of Streptavidin–PE was added to each well and incubated in the dark for 30 min on a plate shaker. Finally, after washing, the microspheres in each well were resuspended in 100 µL wash buffer and shaken for 2 min at room temperature. The plate was read and analyzed on the Luminex 200 analyzer and examined proteins concentrations were determined from seven different standard curves showing median fluorescence intensity vs. protein concentration. 

### 5.4. RNA and MiRNA Isolation

Total RNA enriched in miRNAs was isolated from umbilical cord blood cells (1 × 10^6^) collected from appropriate-for-gestational-age preterm and term newborns using the mirVana™ miRNA Isolation Kit (Thermo Fisher, Waltham, MA, USA) following the manufacturer’s instructions. Concentration and quality of the obtained RNA were assessed by Epoch spectrophotometer (Biotek, Winooski, VT, USA). For subsequent miRNA and whole transcriptome microarray analysis, total RNA enriched in miRNAs isolated from UCB cells samples were pooled to generate one sample per group. 

### 5.5. Affymetrix GeneChip miRNA Microarray 

The procedure using previously described methods [85,86] started with a poly(A) tailing reaction followed by ligation of the biotinylated signal molecule to the target RNA. The next step was sample hybridization onto an Affymetrix miRNA 4.1 Array Strip (Affymetrix). The last step was streptavidin-PE addition and array scanning with Affymetrix GeneAtlas system (Affymetrix). 

### 5.6. Affymetrix GeneChip Whole Transcriptome Microarray

A sense-strand cDNA generated from the total RNA using an Ambion WT Expression Kit (Thermo Fisher Scientific, Waltham, MA, USA) was fragmented and labelled using the GeneChip WT Terminal Labelling Kit (Affymetrix, Santa Clara, CA, USA) and next hybridized onto an Affymetrix Human Gene 2.1 ST Array Strip. The hybridization and subsequent fluidics and scanning steps were carried out with an Affymetrix GeneAtlas System, with designated software. 

### 5.7. Microarrays Data Analysis

Analysis of miRNA and whole transcriptome microarray data was performed using BioConductor software based on the statistical R programming language. The Robust Multiarray Average (RMA) normalization algorithm implemented in the “Affy” library was used for normalization, background correction, and calculation of the expression levels of all of the examined genes and miRNAs.

For miRNA microarray, normalized data were combined with “pd.mirna.4.1” description file, containing, among others, names, types, and sequences of miRNAs. Differential expression was determined by applying the linear models for microarray data implemented in the “limma” library. Normalized miRNA expression datasets were visualized on scatter plots with relation to determined cut-off criteria (fold change higher than |2|). A list of experimentally validated miRNA target genes was downloaded from miRTarBase—a database of mRNA-target interactions. Only targets for differentially expressed miRNA were subtracted from the whole human miRNA-target dataset. A target gene list from each of the comparisons was subjected to functional annotation and clusterization using the DAVID (database for annotation, visualization, and integrated discovery). Target symbols of differentially expressed miRNA were uploaded to DAVID by the “RDAVIDWebService” BioConductor library, where targets were assigned to relevant Gene Ontology (GO) terms.

For whole transcriptome microarray, the normalized data set was merged with an annotated data frame object from the BioConductor “oligo” package, leading to a complete gene data table. The selection criteria for significantly changed gene expression were based on the expression fold difference higher than |2|. Functional annotation clustering of differentially expressed genes was performed using DAVID database and is shown as a bubble plot. The cut-off criteria for generation of bubble plot were as follows: *p-*value < 0.5, adjusted method = Benjamini, and minimal number of genes per group  =  5. Groups of genes fulfilling the mentioned criteria are presented in a graph, in which the bubble size indicates the number of genes represented in the corresponding annotation.

### 5.8. Statistical Analysis

Since the distribution of the quantitative variables was significantly different from the normal distribution in most cases, the nonparametric Mann–Whitney test was used to compare values between groups. *p* < 0.05 was considered statistically significant.

## Figures and Tables

**Table 1:** Characteristics of the study groups. In bold, *p*-value < 0.05 considered statistically significant.

Parameter	Preterm Infants	Term Infants	*p*-Value
Number of subjects	27	52	-
Sex (M/F)	15/12	32/20	-
Length (cm)	46.04 ± 5.71	53.53 ± 3.45	<0.001
Weight (g)	2114.07 ± 648.72	3170.96 ± 520.68	<0.001
Head circumference (cm)	29.93 ± 2.70	34.08 ± 1.76	<0.001
Chest circumference (cm)	27.37 ± 3.31	32.59 ± 2.34	<0.001
Apgar score at 1 min	8.19 ± 1.78	9.31 ± 0.95	0.001
Apgar score at 3 min	8.59 ± 1.50	9.53 ± 0.78	<0.001
Apgar score at 5 min	8.85 ± 1.20	9.65 ± 0.63	<0.001
pH	7.32 ± 0.07	7.32 ± 0.05	0.85
pCO_2_ (mmHg)	46.14 ± 6.69	49.95 ± 8.05	0.02
pO_2_ (mmHg)	25.21 ± 12.64	19.36 ± 13.67	0.002
HCO_3_ (mmol/l)	23.64 ± 2.17	24.84 ± 2.26	0.003
BE (mval/l)	−2.23 ± 1.62	−1.73 ± 1.97	0.49
WBC count (10^3^/µL)	12.52 ± 5.13	19.77 ± 7.81	<0.001
RBC count (10^6^/µL)	4.34 ± 0.48	4.85 ± 0.76	0.001
Haemoglobin (g/dL)	15.96 ± 1.74	16.80 ± 3.71	0.06
Haematocrit (%)	44.43 ± 4.72	47.89 ± 7.06	0.03
Platelets (10^3^/µL)	268.33 ± 61.96	226.50 ± 67.90	0.01
Glucose	45.64 ± 21.32	81.12 ± 8.58	0.004
Maternal age (years)	32.13 ± 5.9	31.16 ± 5.4	0.433

**Table 2:** The profile of cellular miRNA up-regulated in preterm newborns in comparison with control full-term neonates (the fold difference).

No.	miRNA Name	miRNA Type	Absolute Fold Change
1	hsa-miR-3135b	miRNA	3.80
2	hsa-miR-4440	miRNA	3.26
3	hsa-miR-3620-5p	miRNA	2.99
4	hsa-miR-8075	miRNA	2.94
5	hsa-miR-4505	miRNA	2.93
6	hsa-miR-6732-5p	miRNA	2.89
7	hsa-miR-4507	miRNA	2.86
8	hsa-miR-6779-5p	miRNA	2.85
9	hsa-miR-4497	miRNA	2.76
10	hsa-mir-6776	stem-loop	2.59
11	hsa-miR-1587	miRNA	2.57
12	hsa-mir-320e	stem-loop	2.54
13	hsa-miR-4492	miRNA	2.41
14	hsa-miR-3149	miRNA	2.40
15	hsa-miR-3195	miRNA	2.39
16	hsa-miR-6803-5p	miRNA	2.36
17	hsa-miR-595	miRNA	2.35
18	hsa-miR-4508	miRNA	2.32
19	hsa-mir-8075	stem-loop	2.23
20	hsa-miR-3148	miRNA	2.21
21	hsa-miR-130b-3p	miRNA	2.15
22	hsa-miR-602	miRNA	2.14
23	hsa-mir-365a	stem-loop	2.13
24	ENSG00000238388	snoRNA	2.10
25	hsa-mir-6722	stem-loop	2.08
26	hsa-miR-7977	miRNA	2.04
27	hsa-miR-1184	miRNA	2.03
28	hsa-miR-378a-3p	miRNA	2.02
29	hsa-miR-1910-5p	miRNA	2.02
30	hsa-miR-6791-5p	miRNA	2.01
31	hsa-miR-6132	miRNA	2.01
32	hsa-miR-4462	miRNA	2.01
33	hsa-miR-4530	miRNA	2.01
